# Working with hazardous and harmful drinkers: derivation and validation of a model for predicting distinct general practitioners groups

**DOI:** 10.1186/1940-0640-8-S1-A60

**Published:** 2013-09-04

**Authors:** Frederico Rosário, Cristina Ribeiro

**Affiliations:** 1 Primary Health Care Center, Tondela, Portugal; 2 Preventive Medicine Institute, Faculty of Medicine, Lisbon, Portugal

## Introduction

Effectiveness of training in increasing general practitioners’ (GPs) screening and brief intervention rates is influenced by their attitudes towards working with hazardous and harmful drinkers.

## Objective

To determine if GPs attitudes provide evidence of the existence of distinct groups towards working with hazardous and harmful drinkers; to derive and validate a model to predict GPs’ group membership.

## Methods

A randomly selected and representative sample of 234 Portuguese GPs completed the Work Package 4 questionnaire of the ODHIN Project. GPs’ attitudes were measured with the Shortened Alcohol and Alcohol Problems Perception Questionnaire (SAAPPQ). Principal component and hierarchical cluster analysis were conducted to define the optimal group number. Final GP classification was achieved with K-means partitioning. Groups were compared regarding age, years of practice, sex, and type of practice (urban, rural, mixed). Logistic regression analysis was conducted to derive the classification model. The model’s specificity, sensitivity, and overall accuracy were calculated.

## Results

Two groups were found. Group A (N=102, 43.6%) had higher scores in all five SAAPPQ dimensions. The biggest differences were found in self-esteem (2.8±0.3, p<0.001) and satisfaction (2.5±0.2, p<0.001), the smallest in legitimacy (0.7±0.2, p=0.005). Group A GPs were predominantly male (53.6% vs. 38.0%, p=0.02), younger (-3.4±1.2 years, p=0.004) and less experienced (-2.7±1.3 years, p=0.03). No differences were found relating to type of practice (p=0.57). Motivation (OR=3.85), self-esteem (OR=3.20) and adequacy (OR=2.45) were significant (all p<0.001) and incorporated into the final model (Nagelkerke R2=0.788), with an AUC of 0.96 (p<0.001). The model correctly classified 90.9% of the individuals, with 91.3% sensitivity and 90.6% specificity.

## Conclusions

GPs attitudes measured with SAAPPQ provide evidence of the existence of two distinct groups towards working with patients with hazardous and harmful drinking. This knowledge can be useful in future studies aiming to design training programmes tailored to the needs of each physician group.

